# Cardiac tamponade from a giant thymoma: case report

**DOI:** 10.1186/1749-8090-7-14

**Published:** 2012-02-06

**Authors:** Osman Fazlıoğulları, Nazan Atalan, Onur Gürer, Serdar Akgün, Sinan Arsan

**Affiliations:** 1 Department of Cardiovascular Surgery, Medicana Hospitals Bahcelievler, Istanbul, Turkey; 2 Department of Anaesthesiology and Reanimation, Medicana Hospitals Camlica, Istanbul, Turkey; 3 Department of Cardiovascular Surgery, Medicana Hospitals Camlica, Istanbul, Turkey

**Keywords:** Cardiac tumors (includes primary, metastatic), mediastinal tumor (includes thymus), thymectomy, thymoma

## Abstract

Thymoma, the most common neoplasm of the anterior mediastinum especially in adults, accounts for 20-25% of all mediastinal tumors and 50% of anterior mediastinal masses. These tumors are routinely asymptomatic for prolonged periods of time. Pericardial tamponade is a very rare initial manifestation of a thymoma. This report presents a patient who had hemorrhagic pericardial tamponade that likely resulted from the largest symptomatic mixed type (type AB) thymoma described in the literature.

## Introduction

The thymus gland usually is found overlying the pericardium and great vessels in the anterior mediastinum. Tumors of the thymus, namely thymomas, may be of the benign or malignant variety. Of patients with a thymoma, one third to one half are asymptomatic, and one third of patients present with local symptoms related to the tumor encroaching on surrounding structures. Most tumors of the mediastinum are diagnosed accidentally by the routin chest x-rays. Chest pain, cough, dyspnea, myasthenia gravis, and weight loss are observed frequently in symptomatic patients. Pericardial effusion may be present in approximately 20% of cases. However, spontaneous hemorrhagic pericardial effusion is an unexpected clinical feature [1-3]. Tumors that have invaded the cardiac or vascular structures indicate a poor prognosis due to the therapeutic limitations [4]. Here we report a case of giant thymoma with pericardial effusion which has treated by surgery and radiotherapy.

## Case Report

**Figure 1:**
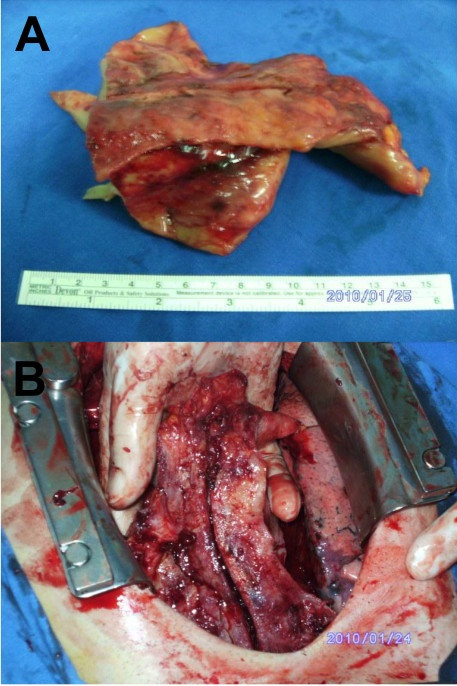
**Mediastinal mass (macroscopic evaluation): A. The line seen in the middle of the mass is from the cutting edge of the saw**. B. The biggest piece of the five pieces was 13 × 10 × 4,5 cm, and the smallest was 3 × 3 × 2 cm. In pictures, only the biggest part can be seen because of its superior portion, 3 cm in diameter, could not be resected due to the invasions of innominate vein and VCS.

**Figure 2:**
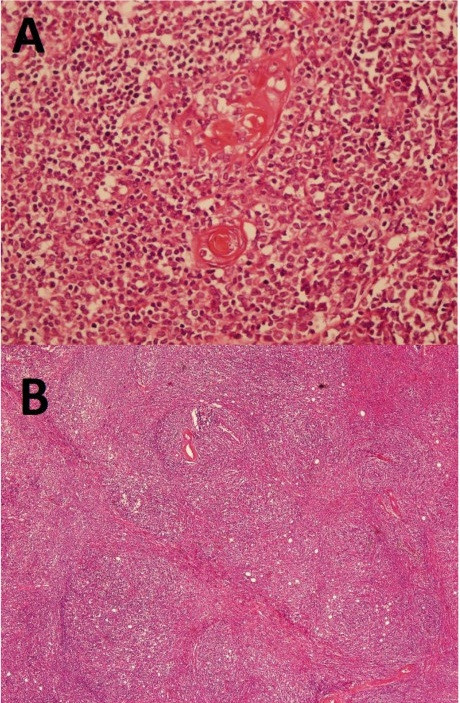
**Histopathological evaluation: A. Massive lymphoid infiltration creating a nodular pattern with fibrous septae**. **B**. Epithelial thymic components in massive lymphoid infiltration.

A 28-year-old man was admitted with sudden onset and severe complaints of orthopnea and palpitations because of a pericardial tamponade resulting from a massive pericardial effusion. He didn't complain any myasthenic symtom like muscular weakness, fatigability, diplopia etc. and trauma in his previous history. The pulse was rapid and weak (142 beats/min), and the neck veins were distended. The electrocardiogram showed sinus tachycardia and low voltage. The blood pressure was 80/60 mmHg. Chest x-ray showed that slightly widened cardio-thoracic index (0.62) and mildly expanded superior mediastinum. Laboratory findings were normal. Echocardiography showed biventricular collapse because of the widespread 4,5-cm pericardial effusion with anterior mediastinal mass. After the evacuation of pericardial hemorrhagic fluid (550 cc), the collapse disappeared, and the patient's symptoms were relieved entirely. The hematocrit value of the pericardial fluid was 36,1%. Cytological and bacterial evaluations were unremarkable. Recurrent pericardial effusion was observed twelve hours later without collapse. Computed tomography (CT) scans showed a huge antero-superior mediastinal mass with rough and longest dimensions of 17 × 12 × 7 cm; the exact dimensions could not be calculated because of the possible invasion into the pericardium, the innominate vein, the superior caval vein (VCS) and the sternum (Figure 1). Surgery was performed via median sternotomy as used frequently for thymic surgical aspects. The tumor's extension into the spaces between pericard, pleura and sternum was seen. Pericardial, pleural and at the superior segment innominate vein were invaded. Approximately 95% of tumor was resected with some parts of pleura and pericard. The superior part of the tumor couldn't resect because of the innominate vein adhesion. The biggest piece of the five pieces which resected has shown in Figure 1. Adhesions and the initial pathological diagnosis, the frozen, was lymphoma did not necessitate a total resection. The mass was off-white in color, solid, hard, and lobulated, and it weighed 640 g. Even the frozen evaluation was lymphoma, the late histopathological evaluation revealed that the mass was type AB according to the World Health Organization (WHO) classification and Stage II according to the Masaoka Staging System (Figure 2). The patient was discharged on the fifth postoperative day. Combined chemotherapy (cisplatin and ifosfamide) and radiation therapy (5000 cGy) was started one month later. CT scan did not reveal any metastasis at the first postoperative month.

## Discussion

Hyperplasia, thymoma and hematopoietic neoplasms are the most common causes of thymic enlargement. The overall incidence of thymoma is rare, 0.15 cases per 100.000 [5]. Histologically, 70-80% of thymomas are benign, but 2-6% of the tumors are thymic carcinomas with high degrees of malignancy. According to the recently-developed WHO classification, type A, AB and B tumors are not categorized as malignant, but type C tumors are considered to be malignant. The size, resectability and invasion of the surrounding structure are the most reliable prognostic factors for Masaoka staging [2,4,5]. Suspected malignancy and the presence of myasthenia gravis are surgical indications [4]. Total resection should be verified with frozen sections during surgery for the removal cysts, hyperplasia and thymic tumors, except for lymphomas, seminomas and malignant germ cell tumors [4-7].

Thymomas account for approximately 20-25% of all mediastinal masses in people of all ages and 47% of all mediastinal masses in adults. These tumors are frequently asymptomatic [1-3]. Although only one-third of patients with localized disease are symptomatic, most patients with disseminated disease have significant complaints, such as chest pain, chest discomfort, dyspnea, and superior vena cava syndrome [1-5]. However, hemorrhagic pericardial tamponade is an uncommon initial manifestation.

Because complete resection and the type of the tumor are the most important factors determining prognosis, the resection should be performed with regard to pathological evaluation for thymic enlargements as much as possible. In this case, because of the size and the dissemination characteristics that were revealed by the CT scans, pericardiectomy and resection were unavoidable. Other diagnostic techniques, such as needle biopsies or VATS, were not used. Because of the pathological similarities between lymphoma and thymoma, we resected the tumor as much as possible, but according to the frozen sections, we did not achieve total resection. Additionally, lymphoma is more likely in this patient because of his age.

## Conclusions

The presence of hemorrhagic pericardial tamponade without any history of chest trauma or any coagulation abnormality, thymic pathologies should definitly be suggested at first sight. Also, to the best of our knowledge, this thymoma is the largest one reported that has been diagnosed based on the sudden onset hemorrhagic pericardial tamponade as an initial symptom. We believe that CT scanning should definitely be performed for all pericardial effusions with unknown ethiology. If thymoma is a possible diagnosis, the first aim in surgical therapy should be total resection since the heterogenity of thymomas pathologic structure. Histological heterogenity of thymomas may cause misdiagnosis since some parts involve lymphocitic and some parts thymic dominancy as in our case.

## Competing interests

The authors declare that they have no competing interests.

## Authors' contributions

MSB drafted the manuscript. OG conceived the study and participated in its design and coordination. AK collected data about the patient. YY participated in the patient follow-up. AÇ participated in the study design and coordination. All authors read and approved the final manuscript.

## Consent statement

Written informed consent was obtained from the patient for publication of this case report and accompanying images. A copy of the written consent is available for review by the Editor-in-Chief of this journal.
